# Autoalgometry: An Important Tool for Pressure Pain Threshold Evaluation

**DOI:** 10.3390/jcm7090273

**Published:** 2018-09-12

**Authors:** Letizia Lorusso, Monica Salerno, Francesco Sessa, Daniela Nicolosi, Lucia Longhitano, Carla Loreto, Marco Carotenuto, Antonietta Messina, Vincenzo Monda, Ines Villano, Giuseppe Cibelli, Anna Valenzano, Marcellino Monda, Paolo Murabito, Maria Pina Mollica, Giovanni Messina, Andrea Viggiano

**Affiliations:** 1 Department of Medicine, Surgery and Dentistry “Scuola Medica Salernitana”, University of Salerno, 84081 Baronissi, Italy; liveflame@hotmail.it (L.L.); aviggiano@unisa.it (A.V.); 2 Department of Clinical and Experimental Medicine, University of Foggia, 71122 Foggia, Italy; monica.salerno@unifg.it (M.S.); francesco.sessa@unifg.it (F.S.); giuseppe.cibelli@unifg.it (G.C.); anna.valenzano@unifg.it (A.V.); 3 Department of Biomedical and Biotechnological Sciences, University of Catania, 95131 Catania, Italy; danielanicolosi03@gmail.com (D.N.); lucialonghitano@hotmail.it (L.L.); carla.loreto@unict.it (C.L.); 4 Clinic of Child and Adolescent Neuropsychiatry, Department of Mental Health, Physical and Preventive Medicine, University of Campania “Luigi Vanvitelli”, 80138 Naples, Italy; marco.carotenuto@unicampania.it; 5 Department of Experimental Medicine, Section of Human Physiology and Unit of Dietetic and Sport Medicine, University of Campania “Luigi Vanvitelli”, 80138 Naples, Italy; antonietta.messina@unina2.it (A.M.); vincenzo.monda@unicampania.it (V.M.); inesvillano@hotmail.com (I.V.); marcellino.monda@unicampania.it (M.M.); 6 Department of Surgery and Surgical Medical Specialties, University of Catania, 95131 Catania, Italy; paolomurabito@tiscali.it; 7 Department of Biology, University of Naples Federico II, 80138 Naples, Italy; mariapina.mollica@unina.it

**Keywords:** autoalgometry, pain threshold, test speed, gender

## Abstract

The term “pain threshold” refers to the measurement of the intensity of a physical stimulus that evokes pain. To estimate the pain threshold, a mechanical or electrical stimulus with increasing intensity is usually applied until the subject under evaluation refers to a pain sensation. This study aims to evaluate the autoalgometric pain threshold as a perfect technique to determine the effects of stimulation rate in relation to both gender and the site of stimulation. In this experimental model, pressure algometry was applied: the subject under evaluation pushed a finger against a small round metal tip, producing and at the same time controlling the intensity of the noxious stimulus. Through autoalgometry, the stimulus intensity was recorded over time, measuring the force change rate applied and studying the subject’s behavior on approaching pain. This test was performed with 50 healthy volunteers on two days, applying a fast or slow rate of stimulation. The results described demonstrate that there is a positive correlation between the pressure increase rate and the pressure threshold evaluation. In light of these findings, autoalgometry can be proposed as an objective measure of pressure pain threshold for clinical and research use.

## 1. Introduction

Pain has a conservative effect for humans and animals, to avoid harming the body and to search for a remedy if any harm occurs. Pain is also one of the most relevant clinical symptoms; in fact, it is the main reason to ask for medical help. Under some circumstances, pain is itself a pathological condition. For these reasons, there is great interest in understanding the physiological mechanisms that evoke pain, from painful stimulus transduction, transmission, and modulation to subjective perception. This knowledge is necessary to recognize the causes of pain and find the best possible remedy.

As a general definition, pain is a subjective sensation, so it cannot be measured. To evaluate pain in human scientific studies, two methods are usually applied. In the first method, people record their personal evaluation of pain intensity on a hypothetical scale with minimum and maximum values (e.g., rating from 0 to 10 or positioning a cursor on a visual scale). Weerasinghe et al. [1] utilized a pain scale to evaluate pain pressure thresholds in their study on the human plantar surface. In the second method, the intensity of the physical stimulus that evokes pain is evaluated; this is commonly called the pain threshold. Even if a measurement is generated, it is still based on a subjective evaluation. Both methods are important for pain evaluation and are used in clinical practice.

Several studies have described different stimuli to evaluate pain thresholds, such as pressure [1,2,3,4,5,6,7,8,9,10], heat [11,12], electric current [13,14,15,16,17,18], and intra-epidermal injection of chemical substances (e.g., capsaicin [19] or acid type solution at pH 4.3 [20]). Despite the fact that the most abundant nociceptors are polymodal, generally different stimuli activate different nociceptors clusters; for this reason, it is important to study all kinds of stimuli to improve all technical aspects of pain threshold evaluation. One of the simplest and most widely used methods to evaluate pressure pain threshold is the Fischer algometer, which is essentially a graduated dynamometer. An examiner presses the tip against the skin of the evaluated subject with increasing force; the test is stopped when the subject refers to pain, and the examiner records the force applied [3]. More recent variants of this method permit digital force recording; alternatively, the patient can push a button to communicate the pain sensation and eventually have electromechanical control of the algometer tip. In all cases, the evaluated subject has no control over the intensity of the painful stimulus but can only stop it. This kind of algometer is mainly used with a round tip with a surface of 1 cm^2^ to evaluate pressure pain evoked in muscles or over tendon insertions, which is relevant for some pathologies (e.g., fibromyalgia). A similar procedure, commonly referred to as pinprickalgometry, is used with smaller probes (below 1 mm in diameter) to evaluate pain evoked on the skin, which can be preferentially affected in some pathologies (e.g., diabetic neuropathy) [7]. In the case of pinprickalgometry, however, the procedure is longer; considering that various probes of predefined weight are usually applied, the evaluation of the perceived pain intensity for each probe must be recorded.

In an effort to develop a method to evaluate pressure painthreshold on skin that is as simple and fast as Fischer algometry but avoids any external intervention in the control of stimulus intensity, another method, called autoalgometry, has been proposed [21]. During this procedure, the evaluated subject can press one finger against a rounded probe 1 mm in diameter with increasing force until he or she experiences a pain sensation. The probe is vertically mounted on a computerized weighing device that records the force applied during the experiment. In this way, it is possible to evaluate the pressure pain threshold, avoiding errors due to the external maneuvers of a human examiner. Moreover, autoalgometry permits study of the slope of stimulus intensity. This parameter is of interest for at least two reasons: (1) it can be indicative of personal propensity to tolerate pain, and (2) it can affect the threshold favored by the algometric procedure. The latter effect has already been described for the heat-induced pain threshold, in which the threshold increased with the rate of stimulation [10]. This effect has been attributed to an artifact due to the time lag between the activation of nociceptors, the conscious pain sensations, and the motor response to pain. The same effect has been described for pressure algometry with a 0.5 cm^2^ probe [9] and a 3 cm^2^ probe [7].

This study aims to evaluate the autoalgometric pain threshold as a perfect technique to determine the effects of stimulation rate in relation to both gender and the site of stimulation.

## 2. Experimental Section

### 2.1. Subjects

Fifty healthy volunteers (21 men, 29 women, ages 18–29 year) were enrolled. All subjects were informed about the experimental procedure and the study aims. All subjects gave written informed consent in accordance with the latest version of the Declaration of Helsinki. The protocol was approved by the Human Ethical Review Committee (Prot. No. 38862), University of Salerno.

### 2.2. Autoalgometry

**Figure 1:**
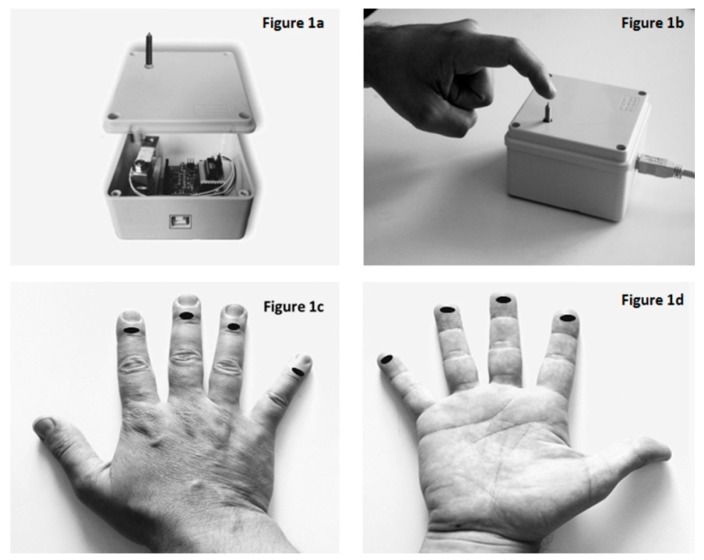
The autoalgometer consists of a cylindrical metal probe 1 mm in diameter with a round tip, fixed in a vertical position to a cell load. (**a**) On the back side, there is a USB port that connects the device to the PC. (**b**) Each subject pushes down on the tip of the probe, increasing pressure (slow or fast) until a minimum or maximum tolerable pain sensation is felt. The test was performed for each hand landmark: eight points on the dorsal surface (**c**) and the tip (**d**) of the third phalanx of the second, third, fourth, and fifth fingers were recorded.

The procedure for pain threshold evaluation was described elsewhere [22]. Briefly, the instrument (autoalgometer) consisted of a cylindrical metal probe 1 mm in diameter with a round tip fixed in a vertical position to a cell load. The signal from the sensor was acquired and stored on a PC over time at a sampling frequency of 10 samples/s. Each subject was asked to slowly push down on the metal probe, increasing the pressure, until he or she felt a minimum pain sensation (minimal test-slow). This test was repeated on 8 points: the tip and the dorsal surface of the third phalanx of the second, third, fourth, and fifth fingers (Figure 1). Then, the subject was asked to repeat the same procedure on the same 8 points but slowly increase the pressure until he or she felt the maximum pain sensation he or she could tolerate (maximal test-slow). On a different day, at least 8 days from the previous session, the same subjects were asked to repeat the procedures on the same 8 testing points, but to increase the pressure quickly until they felt the minimum (minimal test-fast) or maximum (maximal test-fast) tolerable pain. Half of the subjects performed the slowtest in the first session and the fasttest on the other day, while the other half performed the fasttest in the first session and the slowtest on the other day.

### 2.3. Statistical Analysis

Data are presented as mean ± standard error (SE). Correlation of data was evaluated with Pearson’s coefficient (*r*) and the *t*-test on the *r* statistic. Significant differences between mean values were evaluated with analysis of variance (ANOVA) and Tukey’s post hoc test.

## 3. Results

**Figure 2:**
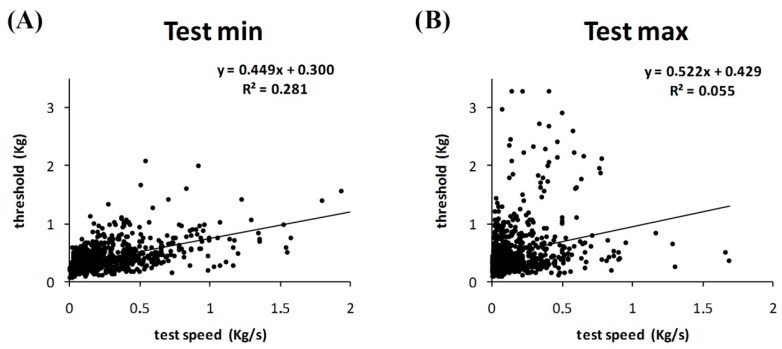
Correlation between speed test and pain threshold. (**A**) A positive correlation is shown between pain threshold and test speed in minimal tests; (**B**) this correlation is lower in maximal tests.

Considering both slow and fast tests performed by all participants, there was a positive correlation between test speed and algometric threshold in the minimal tests (*r* = 0.53, *p* < 0.01), but not in the maximal tests (Figure 2).

**Figure 3:**
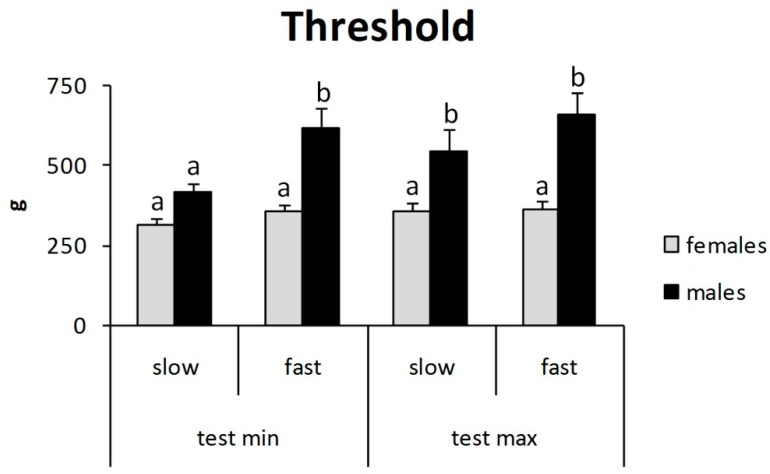
Autoalgometric threshold: gender differences.

For men, the mean algometric threshold was lower in the minimal test-slow then in the other experimental conditions, and in this case, it did not differ from thresholds for women (Figure 3). Contrariwise, for women, there were no significant differences between all experimental conditions (Figure 3). ANOVA demonstrated a significant effect for sex (F_1, 1216_ = 265, *p* < 0.01), experimental condition (F_3, 1216_ = 20, *p* < 0.01), and the interaction of sex with experimental condition (F_3, 1216_ = 12, *p* < 0.01). Tukey’s post hoc test showed that the mean algometric thresholds in the malemaximal-slow, maleminimal-fast, and malemaximal-fast groups were different from the others (Figure 3).

Tukey’s post hoc test showed that mean algometric threshold in the malemaximal-slow, maleminimal-fast, and malemaximal-fast groups were different from the others.

**Figure 4:**
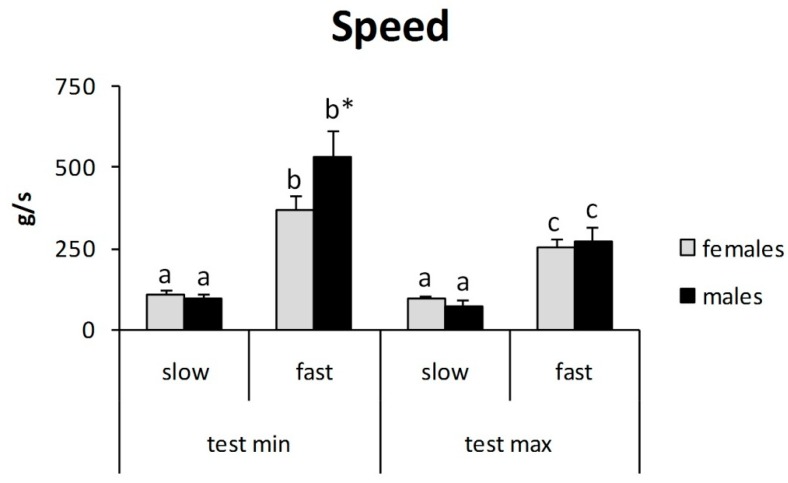
Speed test: difference between fast and slow test. * *p* < 0.01.

Observing the mean test speed for each experimental condition, no differences between male and female participants were observed, while, as expected, it was higher in the fastsessions than in the slowsessions (Figure 4). ANOVA showed a significant difference in evaluating sex (F_1, 1216_ = 12, *p* < 0.01), experimental condition (F_3, 1216_ = 173, *p* < 0.01), and the sex interaction under experimental conditions (F_3, 1216_ = 11, *p* < 0.01). Tukey’s post hoc test showed that mean test speeds in the femaleminimal-slow, maleminimal-slow, femalemaximal-slow, and malemaximal-slow groups were different from the others (Figure 4).

Minimal- and maximal-slow test and maximal-fast test did not differ in speed between men and women; maximal-fast test for menwas faster than for women.

**Figure 5:**
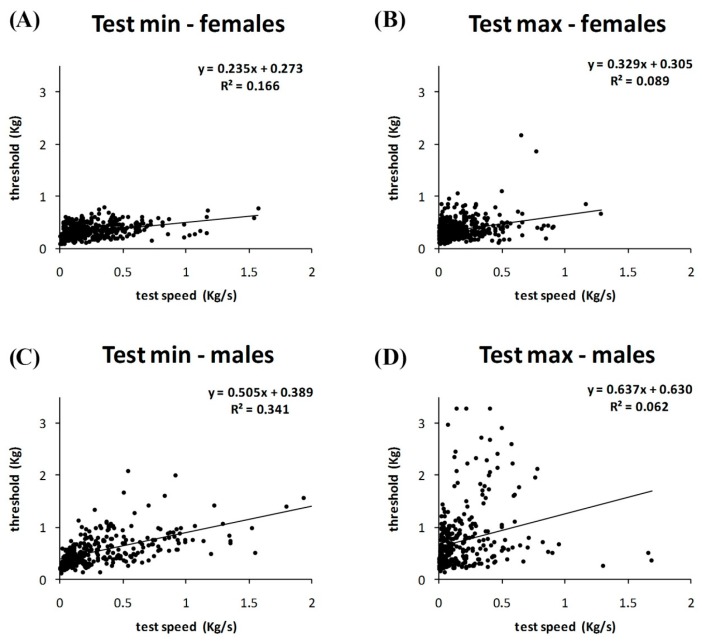
Difference in (**A**,**B**) female and (**C**,**D**) male participants.

The correlation between test speed and algometric threshold was different between male and female participants. The correlation was greater in maleminimaltests (*r* = 0.58, *p* < 0.01) than in femaleminimal tests (*r* = 0.41, *p* < 0.01) or malemaximaltests (*r* = 0.25, *p* < 0.01) or femalemaximaltests (*r* = 0.30, *p* < 0.01) (Figure 5).

Minimal and maximal tests for men and minimal test for women show a positive correlation between pain threshold and test speed.

**Figure 6:**
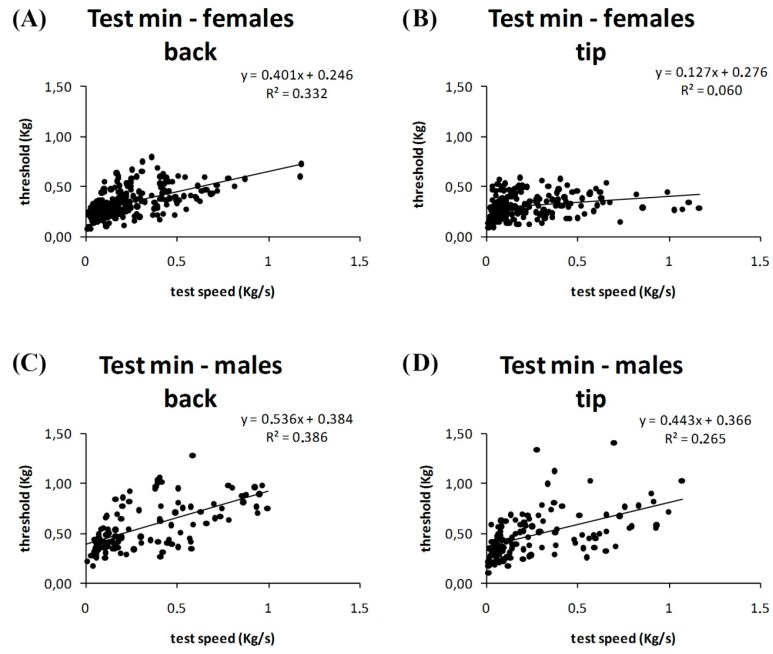
Differences in autoalgometric threshold divided by site of stimulation: (**A**,**B**) female; (**C**,**D**) male.

Further subdivision of the data between values obtained with finger tips and with finger backs revealed that the correlation between test speed and algometric threshold was high even in femaleminimal tests obtained on finger backs (*r* = 0.58, *p* < 0.01), in maleminimal tests obtained on finger backs (*r* = 0.39, *p* < 0.01), and in maleminimal tests obtained on finger tips (*r* = 0.51, *p* < 0.01), but was still low in femaleminimal tests obtained on finger tips (*r* = 0.24, *p* < 0.01) (Figure 6).

In minimal test, the positive correlation between pain threshold and test speed is greater forfinger backs than tips. This correlation remains for women’s and men’s finger backs and men’s finger tips, but not women’s finger tips.

**Figure 7:**
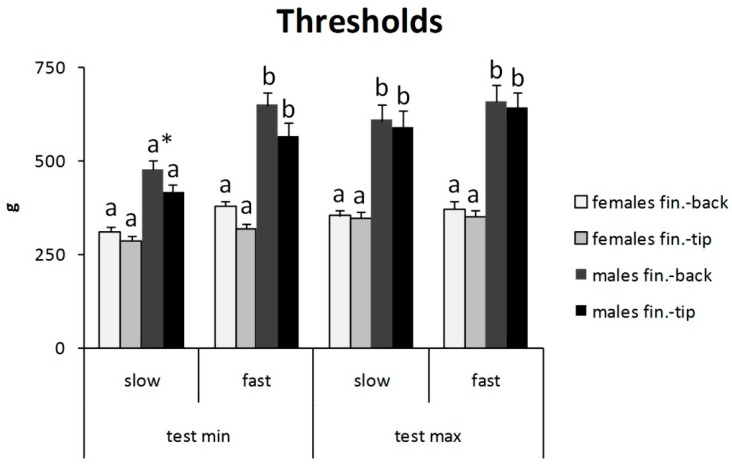
Algometric threshold differencesfor site of stimulation. Fin., finger. * *p* < 0.01.

Considering the same experimental conditions and sex, the mean values for the algometric threshold obtained with finger tips did not differ significantly from those obtained with finger backs. ANOVA showed a significant effect for sex (F_1, 752_ = 221, *p* < 0.01) and experimental condition (F_7, 752_ = 5.99, *p* < 0.01). Tukey’s post hoc test showed that the groups maleminimal-fast (backs or tips), malemaximal-slow (backs or tips), and malemaximal-fast (backs or tips) differed from the others. Moreover, the group maleminimal-slow-backs differed from the group female-minimal-slow-backs, but the group maleminimal-slow-tips did not differ from the group femaleminimal-slow-tips (Figure 6). A subdivision of test speed data between finger tips and backs (Figure 7) did not show other differences than those shown in Figure 4.

The group male minimal-slow differed for backs from female minimal-slow, but not for tips. No relevant differences were seen in speed tests.

## 4. Discussion

The results of the present study demonstrate that the rate of pressure variation is an important variable to consider when evaluating pressure threshold with small probes over the skin. The general trend is to obtain greater autoalgometric values with greater test speeds. These findings are in accord with previous data on heat pain threshold [11] and deep tissue (large probe) pressure pain threshold [8,22,23]. However, the present study also shows that test speed has different effects, depending on: (1) kind of pain threshold (minimal or maximal), (2) gender, and (3) site of stimulation (finger tips or finger backs). It can be argued that other factors influence the pain threshold, maybe more than the test speed. By definition, the maximal pain threshold is the maximal tolerable pain. It is clear that “tolerance” is due to a large extent to personal attitude, rather than to the absolute intensity of the physical stimulus; this can easily explain the absence of a relevant correlation between test speed and maximal pain threshold (Figure 2, Figure 3 and Figure 4). The finding of a lower pain threshold in young women than in young men (Figure 3, Figure 4, Figure 5 and Figure 6) also agrees with the literature data [9,24,25]. Several studies reported that men have significantly higher pressure pain thresholds than women [1].

It was interesting to note that the present results failed to show significant mean different values between minimal and maximal pain thresholds in women (Figure 3, Figure 4, Figure 5 and Figure 6). These findings suggest a very low pain tolerance for young women and/or high sensitivity of skin nociceptors to mechanical stimuli, leading to greatly increased activity over a small variation of pressure; these hypotheses deserve further study. Moreover, it is possible that women express pain while men holdback emotions of pain [1]. This aspect represents an important limitation of this experimental model that is based on the personal identification of pain intensity.

Another unexpected finding was the variable relationship between test speed and minimal pain threshold in women. In particular, in women, a relevant correlation between test speed and minimal pain threshold was observed with finger backs (similar to the correlation observed in men), while such correlation in women was very small with finger tips (Figure 5). This finding suggests possible differences in innervation and mechanical properties of the skin between finger backs and finger tips and between men and women. Differences in skin innervation were previously described: compared to hairy skin, glabrous skin lacks type II A-fiber mechanoheat nociceptors [12]. Moreover, differences in nerve neurophysiology between men and women have been reported; in fact, ulnar and radial nerve conductance velocity seems greater in young women than men [26,27].

The positive correlation between test speed and algometric threshold is probably due to the delay in pain perception and pain reaction. This suggests that a shorter delay in pain reaction should produce a less evident effect of test speed on the algometric threshold. Thus, it could be argued that pain transmission or pain reaction time in women could be faster than in men. This hypothesis has to be addressed in future experiments.

The reaction time to a heat pain stimulus over the hand has been reported on the order of 500 ms (700 ms minus 200–250 ms to reach the desired stimulus intensity) [28]. Because the reaction time to an auditory stimulus is greater than 100 ms, an additional significant delay is expected to occur in the conventional pressure algometry, in which the examiner stops the pressure upon hearing the subject say “Pain.” Thus, it is imperative to pay attention to the rate of stimulation in algometry.In light of these findings evaluating pressure pain thresholds and considering its reliability, the autoalgometer may be an important tool in clinical studies of pain. The autoalgometer could ascertain any changes in pain sensitization in numerous clinical cases, such as hypertension-associated hypoalgesia [21], diabetic neuropathy, neurological diseases that affect pain sensibility, or in functional diseasessuch as fibromyalgia. Furthermore, it could assure better outcomes during follow-up of pharmacology pain therapy or for analgesic management in postoperative neuroplasticity [29]. In this regard, autoalgometry is a convenient method because it avoids any error due to an external examiner and permits the evaluation of the rate of stimulus intensity for the entire time during the test. Moreover, it is very important to note that in clinical use, chronic pain involves a complex interaction of physiological and psychosocial factors, and successful intervention requires the coordinated effort of a treatment team with expertise in a variety of therapeutic disciplines. Psychological evaluations should necessarily include the assessment of sensory, affective, cognitive, and behavioural components of the pain experience and identification of personality and psychosocial factors that can influence treatment outcome.

Others studies should be conducted to validate the variability of pain threshold in the described pathologies, evaluating their progression with the aim to build a cutoff that could predict the speed of progression of the disease.
